# The Effects of External Interfaces on Hydrophobic Interactions I: Smooth Surface

**DOI:** 10.3390/molecules29133128

**Published:** 2024-07-01

**Authors:** Qiang Sun, Yan-Nan Chen, Yu-Zhen Liu

**Affiliations:** Key Laboratory of Orogenic Belts and Crustal Evolution, Ministry of Education, The School of Earth and Space Sciences, Peking University, Beijing 100871, Chinaliuyuzhen@stu.pku.edu.cn (Y.-Z.L.)

**Keywords:** water, hydrogen bonding, hydrophobic interactions, interface

## Abstract

External interfaces, such as the air–water and solid–liquid interfaces, are ubiquitous in nature. Hydrophobic interactions are considered the fundamental driving force in many physical and chemical processes occurring in aqueous solutions. It is important to understand the effects of external interfaces on hydrophobic interactions. According to the structural studies on liquid water and the air–water interface, the external interface primarily affects the structure of the topmost water layer (interfacial water). Therefore, an external interface may affect hydrophobic interactions. The effects of interfaces on hydrophobicity are related not only to surface molecular polarity but also to the geometric characteristics of the external interface, such as shape and surface roughness. This study is devoted to understanding the effects of a smooth interface on hydrophobicity. Due to hydrophobic interactions, the solutes tend to accumulate at external interfaces to maximize the hydrogen bonding of water. Additionally, these can be demonstrated by the calculated potential mean forces (PMFs) using molecular dynamic (MD) simulations.

## 1. Introduction

External interfaces are ubiquitous in nature, such as air–water, oil–water, and solid–water interfaces. They significantly affect the physical and chemical processes of solutes in water, often contrasting with those in bulk solutions. These can be demonstrated by numerous experimental and theoretical studies on the effects of interfaces on protein folding, ion distributions, and chemical reactions. Protein adsorption at oil–water and air–water interfaces is a well-established and widely studied phenomenon [1], such as the aggregation of Aβ peptides at various interfaces [2,3,4,5,6,7]. In fact, the surface aggregation of proteins is often accompanied by structural changes, such as alterations in molecular conformation, nucleation and the growth of aggregates, and the eventual release of these species from the surface to the bulk [1]. Additionally, ion distribution at interfaces is crucial to many fundamental chemical systems and processes [8,9,10,11,12,13,14], and has been attracting a lot of attention. Based on recent studies [15,16,17,18,19,20], larger anions like Cl⁻, Br⁻, and I⁻ tend to prefer the surface of water droplets, whereas F⁻ and alkali cations are more likely to be fully solvated within the bulk of the water droplets. However, the origin of ion adsorption to aqueous interfaces remains a topic of strong debate [21,22,23,24,25,26,27,28,29]. Recently, it has been discovered that many chemical and photochemical reactions are dramatically accelerated at aqueous interfaces compared to the gas phase or bulk water [30]. The field has evolved rapidly since the discovery of “on-water catalysis” [31], which refers to the dramatic acceleration of reactions at the water surface or its interface with hydrophobic media. Hydrophobic interactions are generally considered the fundamental driving forces behind the self-assembly and stability of nanoscale structures in aqueous solutions; therefore, it is necessary to investigate the mechanism of hydrophobic effects, and the effects of external interfaces on hydrophobic interactions.

To explore hydrophobic effects, Frank and Evans [32] introduced the “iceberg” structural model, which was later refined by Kauzmann [33] and other researchers. This model suggests that water molecules form an ordered “cage” around small hydrophobic solutes such as argon or methane. Although numerous studies [34,35,36,37,38,39,40,41,42] have attempted to validate this model, it still remains a topic of debate. According to Stillinger [43], hydrogen bond networks in water can be disrupted at large hydrophobic surfaces, implying that hydrophobic interactions are influenced by the solute’s size. Lum, Chandler, and Weeks (LCW) [44,45,46] provided a quantitative description of the structural and thermodynamic aspects of hydrophobic hydration at different length scales. Based on LCW theory, a crossover from small- to large-scale behavior is expected at the nanometer length scale [46]. In our recent studies [47,48,49], hydration free energy may be dependent on the solute size. With increasing solute size (or concentrations), it can be divided into initial and hydrophobic solvation processes [47,49]. Additionally, different dissolved behaviors are expected for solutes in various processes, such as dispersed or accumulated distributions in water.

In Ball’s works [50,51], water is highlighted as a crucial player in hydrophobic interactions and is considered an active participant in cell biology. Our recent studies [47,48,49] suggest that hydrophobic interactions arise from the structural competition between interfacial and bulk water. Theoretically, the truncation of hydrogen bonds at the external interface significantly impacts the water structure, thereby affecting hydrophobic interactions within the system. According to Vembanur et al. study [52], when the assembly occurs near an extended hydrophobic surface, this leads to the obvious decrease in the desolvation barriers for the assembly of two or more solutes in bulk water. Therefore, dissolved solutes tend to be aggregated at the interface; consequently, extended hydrophobic surfaces could serve as excellent platforms for catalyzing hydrophobically driven self-assembly [52].

According to our recent studies [47,48,49], this work is devoted to understanding the effects of external interfaces on hydrophobic interactions. It is derived that the interface’s effects on hydrophobic interactions may be related to not only the surface molecular polarity but also the geometric characteristics of the interface, such as shape, surface roughness, etc. As a smooth interface is embedded into water, due to hydrophobic interactions, the solutes tend to aggregate at the smooth interface to maximize the hydrogen bonding of water. This is dependent on the solute size (concentrations). Additionally, these can be demonstrated by the calculated PMFs using MD simulations.

## 2. Results and Discussion

### 2.1. Thermodynamic Analysis

As foreign interfaces are embedded into aqueous solutions, the total thermodynamic functions may contain various interaction energies,

(1) $$\Delta G={\Delta G}_{\text{Water-water}}+{\Delta G}_{\text{Solute-water}}+{\Delta G}_{\text{Interface-water}}+{\Delta G}_{\text{Solute-solute}}+{\Delta G}_{\text{Interface-solute}}$$

where ΔG_Solute-water_ and ΔG_Interface-water_ represent the interactions between the solutes and water, and the external interface and water; ΔG_Solute-solute_ and ΔG_Interface-solute_ mean the interactions between the solutes, and the interface and solutes, respectively. Before the solutes are influenced by other solutes (interfaces), they must approach each other (interfaces). Of course, these may be closely related to the structural rearrangement of water molecules. It is important to investigate the structure of water, and the effects of solutes (interfaces) on water structure.

Numerous studies [53,54,55,56,57,58,59,60] have been carried out to elucidate the structure of liquid water. It has been found that different OH vibrational frequencies correspond to various hydrogen-bonded networks in the first hydration shell of a water molecule (local hydrogen bonding) [53,54]. Under ambient conditions, various local hydrogen-bonded networks may be expected for a water molecule, including DDAA (double donor–double acceptor), DDA (double donor–single acceptor), DAA (single donor–double acceptor), DA (single donor–single acceptor), and free OH vibrations [53,54]. These may be affected by temperature, pressure, dissolved salts, and confinement.

According to structural studies [53,54,61] of water and the air–water interface, dissolved solutes predominantly affect the structure of interfacial water. This results in the formation of the solute–water interface due to the disruption of DDAA hydrogen bonding in interfacial water. Therefore, ΔG_Solute-water_ is calculated using the following formula,

(2) $$\Delta G_{\text{Solute-water}}={\Delta G}_{\mathrm{DDAA}}\cdot R_{\mathrm{Interfacial}\;\mathrm{water}\text{/}\mathrm{volume}}\cdot n_{\mathrm{HB}}$$

in which R_Interfacial water/volume_ is the ratio of the interfacial water layer to volume, n_HB_ represents the average number of hydrogen bonds per molecule, and ∆G_DDAA_ denotes the Gibbs free energy of DDAA hydrogen bonding.

**Figure 1:**
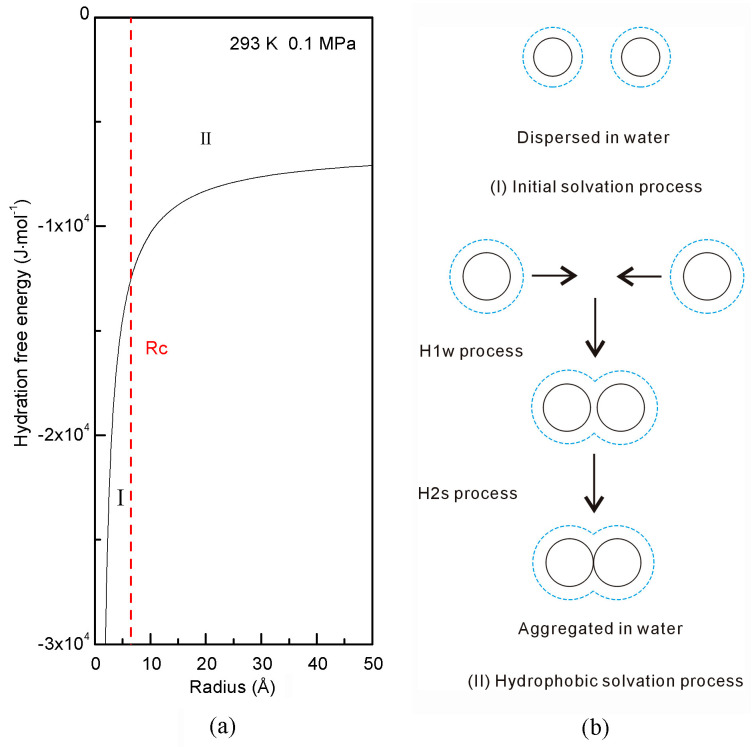
Hydration free energy at 293 K and 0.1 MPa. (**a**) Hydration free energy depends on the solute size. (**b**) Different dissolved behaviors are expected for solutes in initial and hydrophobic processes. As solutes aggregate in water, it is divided into H1w and H2s solvation processes.

When an ideal sphere is embedded in water, hydration free energy is given by (Figure 1),

(3) $${\Delta G}_{\mathrm{Hydration}}={\Delta G}_{\text{Water-water}}+{\Delta G}_{\text{Solute-water}}={\Delta G}_{\text{Water-water}}+\frac{8\cdot {\Delta G}_{\mathrm{DDAA}}\cdot r_{H_{2}O}}{R}$$

where ΔG_Water-water_ and *r*_H_2_O_ mean the Gibbs energy of water [62] and the average radius of a water molecule. A transition is expected when ΔG_Water-water_ equals ΔG_Solute-water_ (Figure 1),

(4) $$\Delta G_{\text{Water-water}}={\Delta G}_{\text{Solute-water}}\;(\mathrm{Rc}=\frac{8\cdot {\Delta G}_{\mathrm{DDAA}}\cdot r_{H_{2}O}}{{\Delta G}_{\text{Water-water}}})$$

where R_c_ represents the critical radius of dissolved solute [47]. At 293 K and 0.1 MPa, R_c_ is 6.5 Å for a spherical solute [48].

Hydration free energy depends on the size of the solute (Figure 1). As the solute size (or concentrations) increases, it is divided into the initial and hydrophobic solvation processes. Additionally, solutes may exhibit different dissolution behaviors in these processes, such as dispersing or accumulating in water (Figure 1). Consequently, hydrophobic effects are expected during the hydrophobic solvation process.

When solutes aggregate in water, the surface area available for interfacial water decreases. Due to the accumulation of solute surfaces in water, the Gibbs free energy of interfacial water can be expressed as follows,

(5) ΔGInterfacial water=γ⋅ΔGSolute-water γ=Surface areaVolumeAggregateSurface areaVolumeNon-aggregate=f1rSeparation

where γ is the geometric factor reflecting changes in surface area. When solutes come into contact in solution, the solute–solute separation is the hydrophobic radius (R_H_) [48]. The hydrophobic solvation process during solute aggregation is divided into H1w and H2s processes [48]. In the H1w process (γ = 1), water molecules are present between the solutes; in the H2s process (γ < 1), the solutes come into contact, reducing the surface area available for interfacial water.

Additionally, while solutes aggregate in water, hydrophobic interactions are related to the number of water molecules changed from the interfacial layer to the bulk water,

(6) $$\begin{array}{ll} \Delta G_{\mathrm{Hydrophobicity}} & =\gamma \cdot {\Delta G}_{\mathrm{Interfacial}\;\mathrm{water}}-\sum_{i=1}^{m}{\Delta G}_{\mathrm{Solute},\text{i-water}}\\ & =n_{\mathrm{Interfacial}\to \mathrm{bulk}\;\mathrm{water}}\cdot {\Delta G}_{\mathrm{DDAA}} \end{array}$$

where n_Interfacial→bulk water_ represents the number of water molecules transformed from interfacial to bulk water as the solutes associate in solution.

When an external interface is introduced into the solution, the Gibbs energy of interfacial water may include contributions from ΔG_Interface-water_ and ΔG_Solute-water_. This suggests that the external interface can affect the structure of interfacial water, thereby influencing hydrophobic interactions. These effects may be observed through changes in the dissolved behavior of solutes in aqueous solutions.

**Figure 2:**
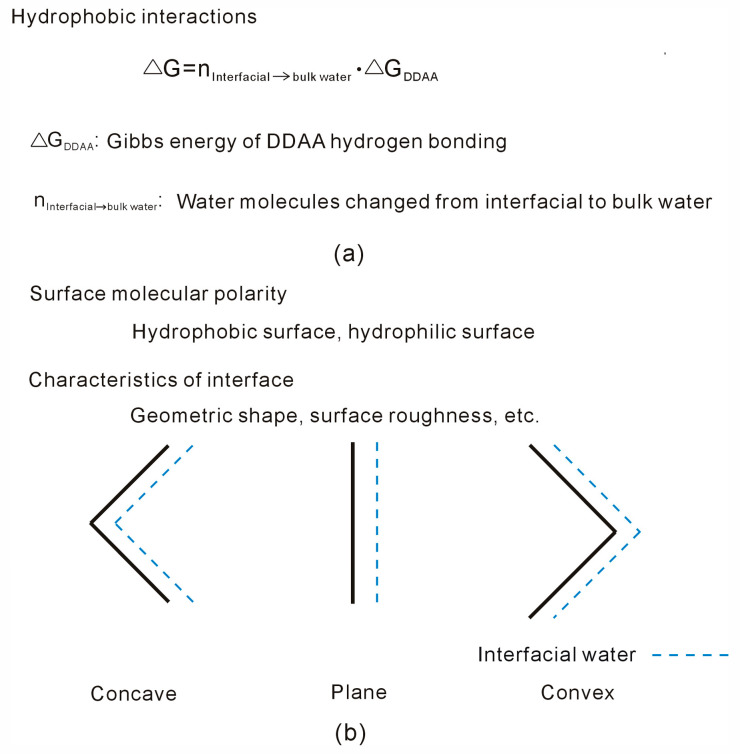
The effects of external interfaces on hydrophobic interactions. (**a**) Hydrophobic interactions are related to water molecules transformed from interfacial to bulk water and Gibbs energy of DDAA hydrogen bonding. (**b**) The effects of external interface on hydrophobic interactions may be related to the surface molecular polarity, and the geometric characteristics of external interface, such as shape, surface roughness, etc.

At 293 K and 0.1 MPa, the R_c_ for two identical spherical solutes is determined to be 3.25 Å [48]. It is widely recognized that the external interface is significantly larger than the solute size. Taking into account the geometric size of the external interface, hydrophobic interactions between the solute and interface can be viewed as an “attractive” force. Due to these hydrophobic interactions, dissolved solutes tend to associate with the external interface to maximize water’s hydrogen bonding. According to Equation (6), as solutes aggregate at the interface, the strength of hydrophobic interactions is closely tied to ΔG_DDAA_ and n_Interfacial→bulk water_. This framework can be used to analyze the effects of external interfaces on hydrophobic interactions (Figure 2).

Based on a vibrational sum frequency generation (SFG) study on the air–water interface [61], the absence of DDAA hydrogen bonding in interfacial water is related to the formation of the air–water interface. In Stillinger’s work [43], the liquid water interface adjacent to an extended non-polar hydrophobic substrate shares the same microscopic features as a liquid–vapor interface. Additionally, it is also supported by the subsequent theory and molecular simulations [63,64]. In fact, it is noted that the Gibbs energy of DDAA hydrogen bonds (ΔG_DDAA_), as expressed in Equation (6), is due to the difference in Gibbs energy between interfacial and bulk water. The effects of an external interface on interfacial water may be influenced by the interactions between them. Therefore, when the external surface is embedded into water, hydrophobic interactions may be affected by the surface molecular polarity of the external interface (Figure 2).

Additionally, based on Equation (6), hydrophobic interactions are related to the number of water molecules transformed from interfacial to bulk water when the solutes are associated with the external interface. To enhance hydrophobic interactions, the solutes tend to be associated with the external interface in a specific direction so that more interfacial water molecules may be changed into bulk water. Therefore, hydrophobic interactions are dependent on the geometric characteristics of the external interface, such as shape (concave, plane, or convex) and surface roughness (Figure 2). Additionally, it is derived that n_Interfacial→bulk water_ is also influenced by the solute shape, size, and concentrations.

**Figure 3:**
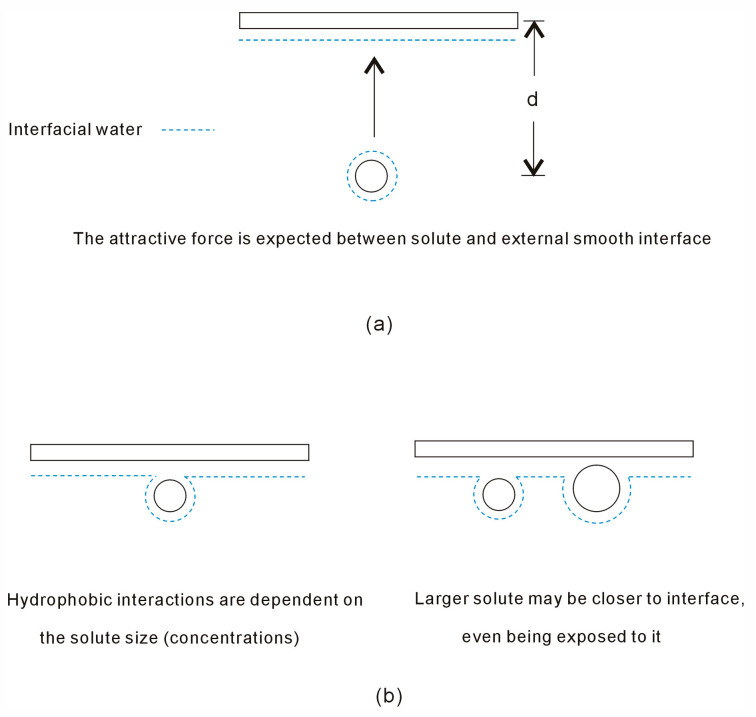
The influence of smooth interfaces on hydrophobic interactions. (**a**) When the solute is associated with the interface, hydrophobic interactions may be related to the separation between them. (**b**) Owing to hydrophobic interactions, the solutes tend to be aggregated with the smooth interface. These may be related to the solute size (concentrations).

This study is focused on the effects of smooth hydrophobic interfaces on hydrophobic interactions. As a smooth interface is embedded into solutions, hydrophobic interactions between the interface and a sphere solute may be related to the separation between them (Figure 3). With decreasing the distance between them, hydration free energy is expressed as,

(7) $${\Delta G}_{\mathrm{Hydration}}={\Delta G}_{\text{Water-water}}+\iint {\Delta G}_{\text{Solute-interface}}\mathrm{dxdy}$$

where ΔG_Solute-interface_ is the Gibbs energy of interfacial water arising from the solute and interface, which is inversely proportional to the distance between them,

(8) $${\Delta G}_{\text{Solute-interface}}\propto 1\text{/}d_{\text{Solute-interface}}$$

where d_Solute-interface_ is the separation between the solute and interface (Figure 3).

To maximize hydrogen bonds, dissolved solutes often associate with external interfaces instead of staying in bulk solutions. This results in solutes being more concentrated at the interface, as opposed to the bulk solution. Theoretical and experimental studies [15,16,17,18,19,20] indicate that larger ions tend to adsorb preferentially to the interfacial region, leading to higher concentrations there compared to the bulk. This behavior can be explained by the influence of external interfaces on hydrophobic interactions.

Dissolved solutes mainly impact the hydrogen bonding of interfacial water. To strengthen hydrophobic interactions, more interfacial water may be converted into bulk water. The influence of external interfaces on hydrophobic interactions is connected to the size and concentration of the solutes (Figure 3). It has also been inferred that larger solutes, as opposed to smaller ones, are more likely to be near the interface and may even be exposed to it.

Due to hydrophobic interactions, the solute tends to approach the external interface or other solutes. As the separation between solute and interface (or between solutes) decreases, the direct interactions between them become stronger, especially when their surfaces come into contact during the H2s process. Thus, the solute distribution at the interface may also be influenced by these direct interactions. Consequently, solute distribution can be reasonably divided into global and local distributions. In other words, global solute distribution is closely related to hydrophobic interactions, while local solute distribution is modulated by direct intermolecular interactions, such as van der Waals forces, etc. These may be applied to understand the mechanism of Hofmeister effects. Further study may be covered in our following work.

In theory, as an external interface is embedded into water, it undoubtedly affects the structure of interfacial water; therefore, the external interface affects the hydrophobic interactions of the system. In combination with our recent studies [47,48,49], it is derived that the dissolved solutes tend to be aggregated at the interface to maximize the hydrogen bonding of water, which may be in contrast with bulk solutions. Additionally, the tendency may also be influenced by the solute size (concentrations).

### 2.2. MD Simulations

Based on our recent studies [47,48], hydrophobic interactions are dependent on the size of the solute. With increasing the solute size, it is divided into the initial and hydrophobic solvation processes; different dissolved behaviors of solutes are expected in various processes. Fullerene has been attracting much attention [65]. In this study, CH_4_-CH_4_ and C_60_-C_60_ are simulated to understand the dependence of hydrophobicity on solute size. Additionally, to investigate the effects of external smooth interface on hydrophobic interactions, graphene is embedded into the solutions. Both graphene–CH_4_ and graphene–C_60_ systems are simulated to understand the dependence of hydrophobic interactions on the solute size in the presence of a graphene interface. Moreover, MD simulations are also conducted on graphene–CH_4_-CH_4_ and graphene–C_60_-C_60_ systems. Compared to CH_4_-CH_4_ and C_60_-C_60_ systems, these may be used to elucidate the effects of graphene interface on hydrophobic interactions within the systems.

**Figure 4:**
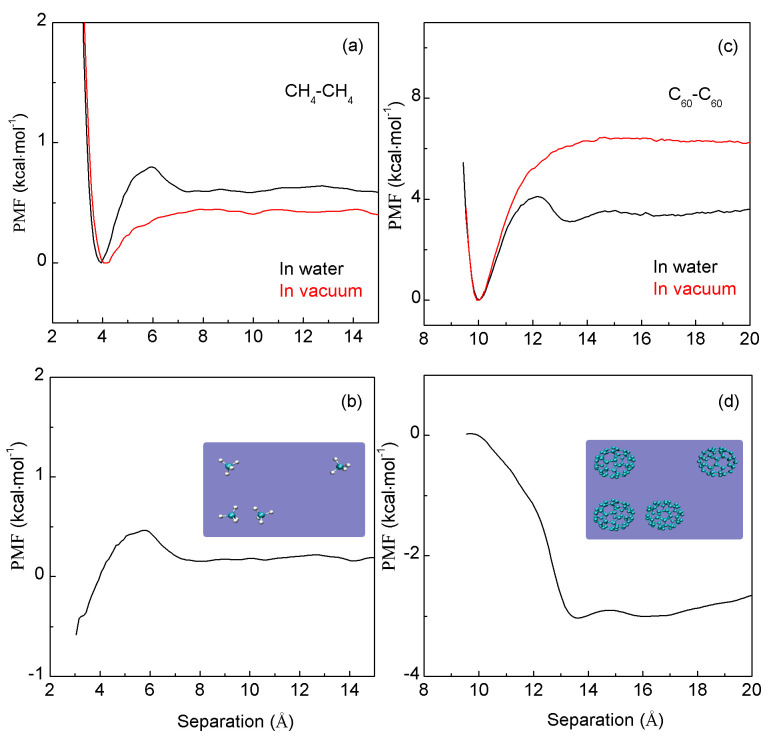
(**a**,**c**) The PMFs when two CH_4_ molecules (two C_60_ fullerenes) are associated in water and in vacuum. (**b**,**d**) Based on the MD simulations, the water-induced PMFs are determined.

In this work, the PMFs are calculated using umbrella sampling (US) method. When two C_60_ fullerenes are associated in water, three minima are found in the calculated PMFs (Figure 4). The first minimum, located at 10.0 Å, represents the contact minimum. The second minimum, positioned at 13.0 Å, represents the solvent-separated PMF, indicating that only one water molecule layer may be found between the two C_60_ fullerenes. A third minimum, at 16 Å, indicates the presence of a double water molecule layer between them. Similarly, three minima are also found in the PMFs between a pair of methane molecules in water. The first minimum is located at 3.9 Å, the second at 6.9 Å, and the third at 10.6 Å (Figure 4). These are in agreement with other MD simulations [48,66,67,68,69,70,71] on C_60_-C_60_ and CH_4_-CH_4_ in water.

Furthermore, as the separation between the C_60_-C_60_ fullerenes (or a pair of methane molecules) decreases, energy barriers are observed in the calculated PMFs between adjacent minima (Figure 4). These barriers stem from the expulsion of a single water layer within the region between solutes as they approach each other. This indicates that the water molecules situated between the solutes are progressively expelled into the bulk water layer by layer as the solutes are brought closer together. Therefore, the energy barriers are associated with the number of water molecules expelled as the solutes are associated in water.

To investigate the hydrophobic interactions resulting from solute–solute association in water, the PMFs between two C_60_ fullerenes (between two CH_4_ molecules) are also calculated in a vacuum (Figure 4). From these calculations, the water-induced contributions (ΔG_Water-induced_) can be determined as the solutes are associated in water,

(9) $${\Delta G}_{\text{Water-induced}}={\Delta G}_{\mathrm{Total}}-{\Delta G}_{\mathrm{Solute}\;\mathrm{in}\;\mathrm{vacuum}}$$

Based on the calculated water-induced PMFs between two C60 fullerenes (two CH_4_ molecules), these can be applied to understand the hydrophobic interactions when they are aggregated in water.

From Figure 4, the water-induced PMFs between two C_60_ fullerenes may be different from those between a pair of CH_4_ molecules in water. Therefore, hydrophobic interactions may be dependent on the size of the solute. As two same-sphere solutes are dissolved into ambient water, R_c_ is 3.25 Å [48]. The radius of CH_4_ (1.9Å) is smaller than R_c_, while the size of C_60_ (5.0 Å) is larger than R_c_ (Figure 4). Therefore, initial and hydrophobic solvation processes are, respectively, expected for CH_4_-CH_4_ and C_60_-C_60_ in water. In other words, it seems that “repulsive” (or “attractive”) forces exist between CH_4_-CH_4_ (or C_60_-C_60_) molecules when they are associated in water.

**Figure 5:**
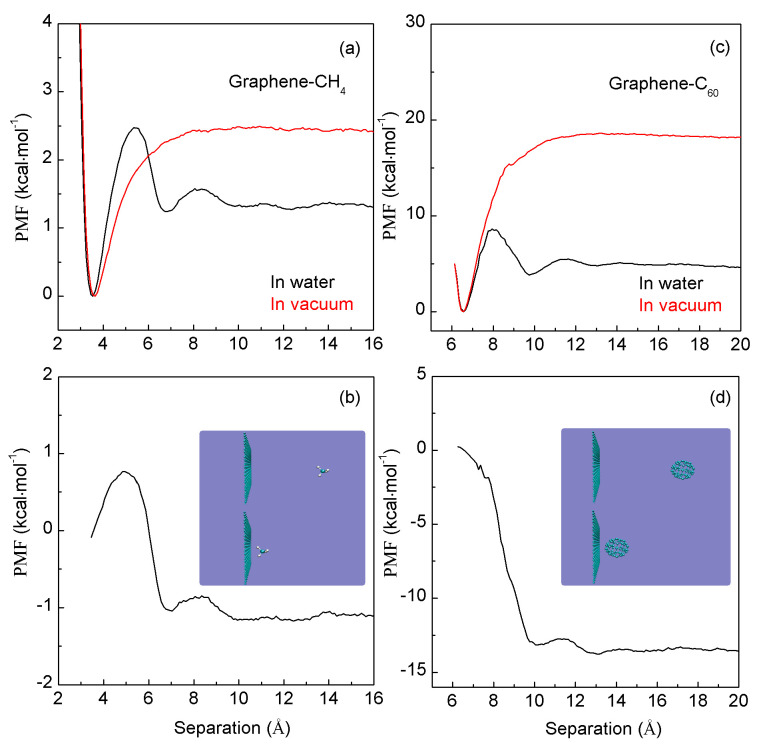
(**a**,**c**) The PMFs when a CH_4_ molecule (a C_60_ fullerene) is associated with graphene sheet in water and vacuum. (**b**,**d**) Based on the calculated water-induced PMFs, they are used to understand the effects of external interfaces on hydrophobic interactions.

To understand the effects of a smooth interface on hydrophobic interactions, a graphene sheet is embedded into the solutions. When a CH_4_ (or C_60_) is associated with the graphene sheet (Figure 5), the PMFs are calculated. In comparison with CH_4_-CH_4_ (C_60_-C_60_) PMFs, a deeper first minimum and a higher first barrier may be found in the PMFs of graphene–CH_4_ (graphene–C_60_) (Figure 5). These differences are attributed to the effects stemming from the graphene.

In this work, to investigate the hydrophobic interactions when CH_4_ (C_60_) is associated with the graphene sheet, the corresponding PMFs in vacuum are also determined (Figure 5). Based on the calculated water-induced PMFs, the “attractive” forces are found in graphene–CH_4_ and graphene–C_60_ systems (Figure 5). This is in contrast with the “repulsive” force between a pair of methane molecules (Figure 4). Therefore, when an interface is embedded into water, the CH_4_ (C_60_) may tend to aggregate with the graphene surface. Additionally, in comparison with graphene—CH_4_, stronger hydrophobic interactions are found between C_60_ and graphene (Figure 5). Therefore, when the solute is associated with the smooth interface, hydrophobic interactions may also be dependent on the solute size.

**Figure 6:**
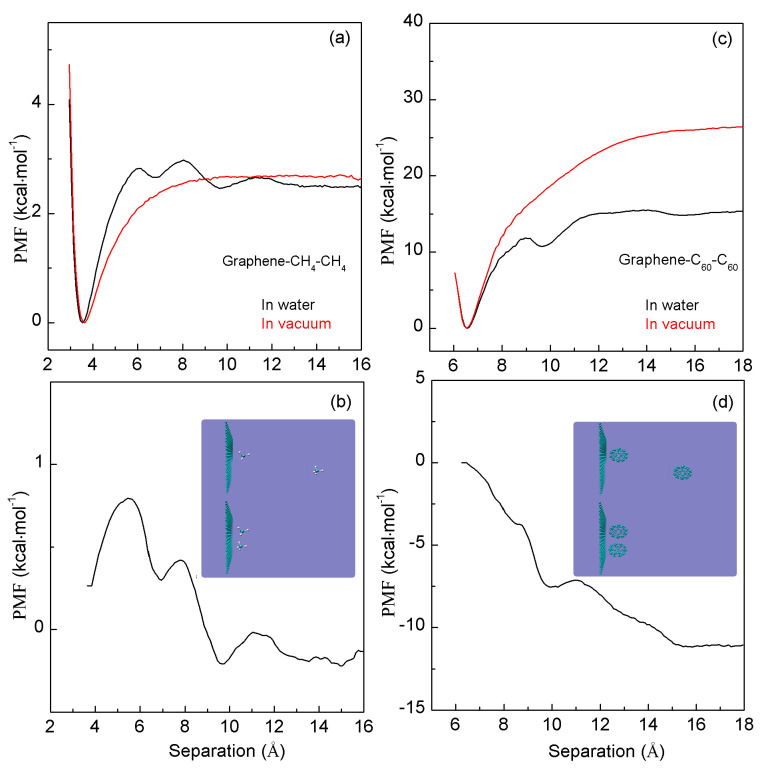
(**a**,**c**) The PMFs when a CH_4_ molecule (a C_60_ fullerene) is associated with graphene–CH_4_ (graphene–C_60_) in water and vacuum. (**b**,**d**) Based on the calculated water-induced PMFs, they are used to study the dependence of hydrophobic interactions on solute concentrations in the presence of graphene.

Additionally, MD simulations are conducted to investigate the dependence of hydrophobic interactions on solute concentrations as they are aggregated at the external interface. In these simulations, two CH_4_ molecules (two C_60_ fullerenes) are sequentially restrained to move toward the center of the graphene, facilitating their aggregation at the surface of graphene (Figure 6). In comparison with the PMFs of graphene–CH_4_ (graphene–C_60_) systems (Figure 5), a deeper first minimum and a weaker first barrier are observed when the second CH_4_ (C_60_) molecule is associated with graphene–CH_4_ (graphene–C_60_) (Figure 6).

In addition, the corresponding PMFs in vacuum are also calculated when the second CH_4_ (C_60_) molecule may be aggregated with the first one at the graphene surface (Figure 6). From these, the corresponding water-induced PMFs (ΔG_Water-induced_) are also determined when CH_4_ (C_60_) is aggregated with graphene–CH_4_ (graphene–C_60_). In comparison with the water-induced PMFs of graphene–CH_4_ (graphene–C_60_) in water (Figure 4), the “attractive” forces are also expected as the second CH_4_ (C_60_) is associated with the first one at the graphene interface. 

**Figure 7:**
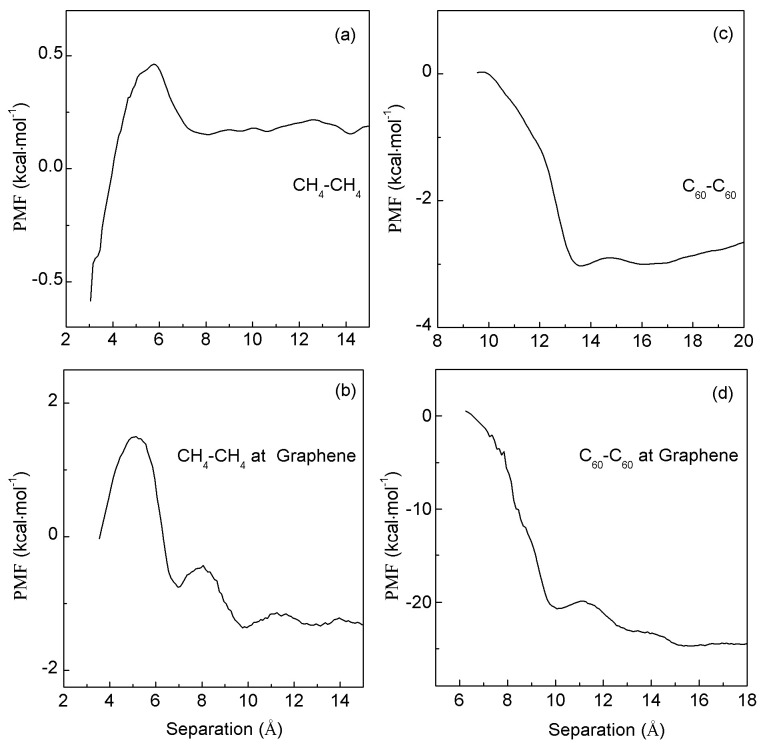
The water-induced PMFs when two CH_4_ molecules (two C_60_ fullerenes) are associated in water (**a**,**c**) and at the graphene surface (**b**,**d**).

Based on the MD simulations, the water-induced PMFs may be determined when two CH_4_ molecules (two C_60_ fullerenes) are associated with the graphene sheet (Figure 7). These may be utilized to understand the hydrophobic interactions arising from the graphene interface. From the simulations, hydrophobic interactions may be influenced by graphene sheets, and strong “attractive” forces are found between solutes and the graphene.

From this work, when the smooth interface is embedded into water, “attractive” forces are expected between the solute and interface. In addition, it is found that hydrophobic interactions may be dependent on the solute size (Figure 5), and solute concentrations (Figure 6). Increasing the solute size (or concentrations) leads to an increase in hydrophobic interactions. Of course, this is also in agreement with the thermodynamic analysis of the effects of external interfaces on hydrophobic interactions.

In the MD simulations, as a graphene sheet is embedded into water, it undoubtedly affects the hydrophobic interactions of CH_4_-CH_4_ (or C_60_-C_60_) in water. Additionally, these may be reflected in the dissolved behaviors of solutes in water. In other words, to be more thermodynamically stable, the two CH_4_ (two C_60_) may be aggregated and distributed near the graphene sheet. In fact, this can be attributed to the tendency of solutes to be aggregated to minimize their surface area-to-volume ratio when they are associated with interfaces.

**Figure 8:**
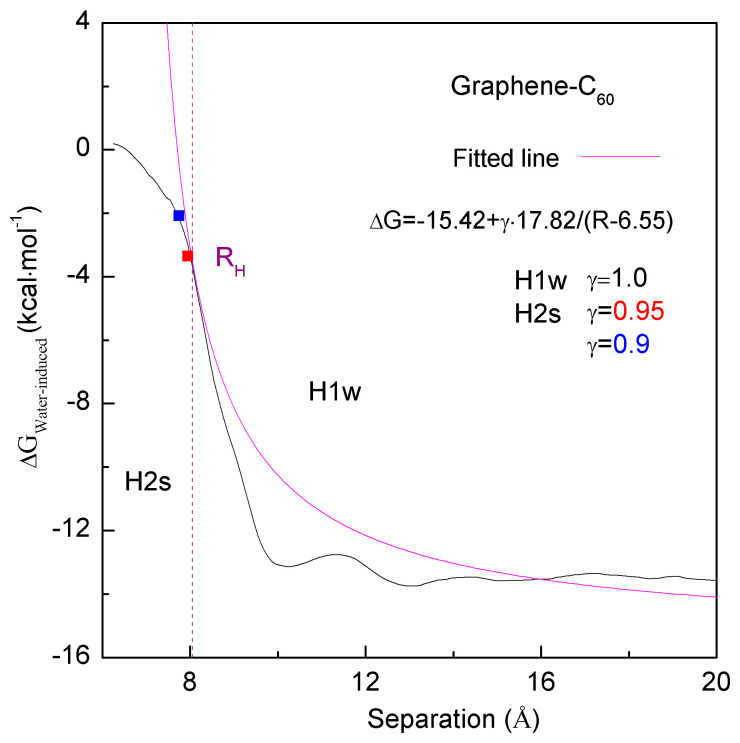
Hydrophobic interactions when a C_60_ fullerene is aggregated with graphene. Hydrophobic interactions are related to the separation between them. The fitted line is shown. When a C_60_ fullerene is associated with graphene, with reference to R_H_, it is divided into H1w and H2s processes.

From the above discussion, when the C_60_ fullerene is associated with the graphene, the hydrophobic interaction is dependent on the separation between them. Therefore, the calculated water-induced PMFs may be reasonably fitted as a function of graphene–C_60_ distance (Figure 8),

(10) $$\Delta G_{\text{Water-induced}}=a+\gamma \cdot b\text{/}\left(R-6.55\right)$$

where R is the separation between the sheet and C_60_ fullerene centers; a and b are fitted to be −15.42 and 17.82, respectively. Additionally, as the fullerene comes into contact with the graphene sheet in water, the separation (R_H_) between them is determined to be 8.05 Å. When the C_60_ is aggregated with the graphene in water, it may be divided into H1w and H2s processes.

When three-dimensional hydrogen bonds form in water, the Raman OH vibrations are predominantly influenced by the local hydrogen bonding of individual water molecules [53,54]. Therefore, under ambient conditions, dissolved solutes primarily impact the structure of interfacial water. This finding aligns with other studies [72,73,74,75,76] on the structure and dynamics of water surrounding ions, suggesting that the influence of ions on water structure is mostly limited to the first solvation shell.

**Figure 9:**
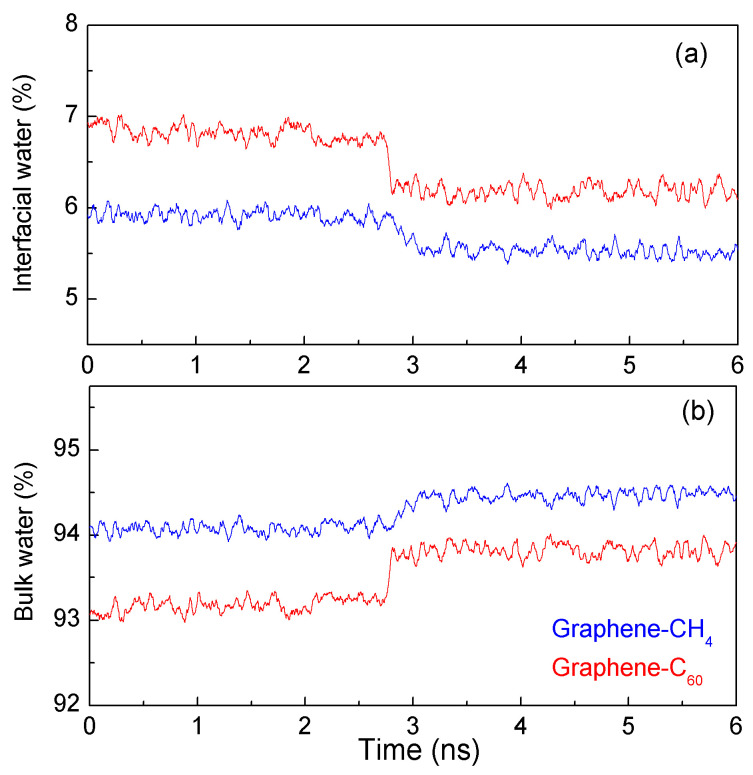
The changes of interfacial (**a**) and bulk water (**b**) when CH_4_ (C_60_) is aggregated with graphene.

According to the MD simulations, both interfacial and bulk water were analyzed as CH_4_ (or C_60_) associated with the graphene sheet in water (Figure 9). During the H1w hydrophobic process, there were no significant changes observed in either interfacial or bulk water; however, when the solute surface made contact with graphite in the H2s hydrophobic process, a noticeable decrease in interfacial water (or an increase in bulk water) was observed. This is attributed to the transition of water molecules from the interfacial layer to the bulk phase. Additionally, compared to the graphene–CH_4_ system, more interfacial water molecules were expelled into the bulk phase as C_60_ associated with graphite (Figure 9). This suggests that the hydrophobic interactions are stronger when C_60_ is associated with graphite compared to CH_4_ (Figure 5).

**Figure 10:**
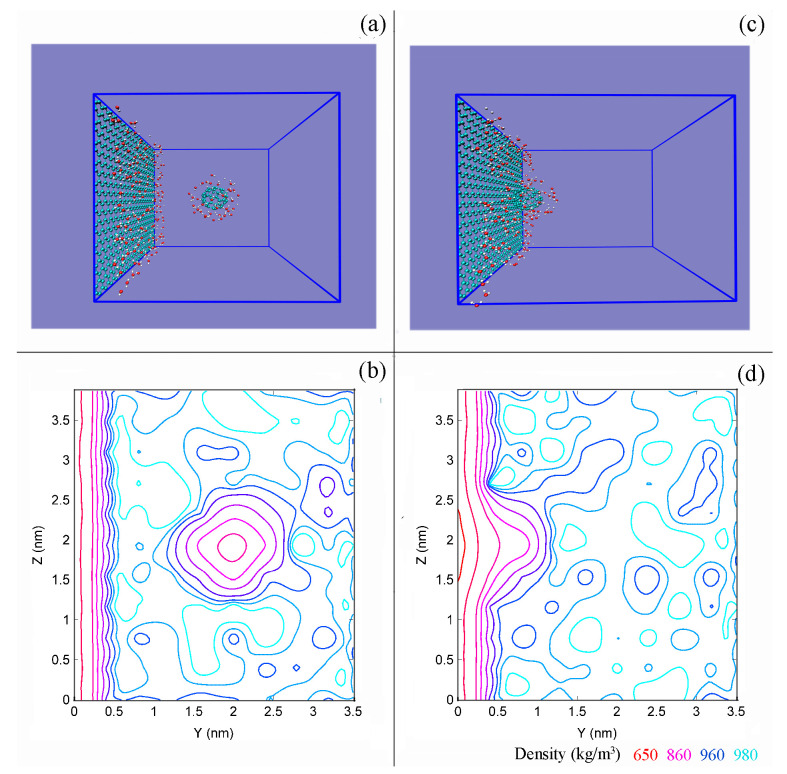
The density of water before (**a**,**b**) and after (**c**,**d**) a C_60_ is associated with graphene sheet. (**a**,**c**) Only interfacial water layers are shown. (**b**,**d**) Various colors are used to show density distributions.

Using MD simulations, the water density distribution was analyzed during the aggregation of solutes with the graphite sheet (Figure 10). Higher density was observed in the interfacial water layer compared to bulk water. This result is consistent with experimental measurements [77] and theoretical simulations [78] on confined water, indicating a structural difference between interfacial and bulk water. This difference may be linked to the formation of DA hydrogen bonds in interfacial water [49].

**Figure 11:**
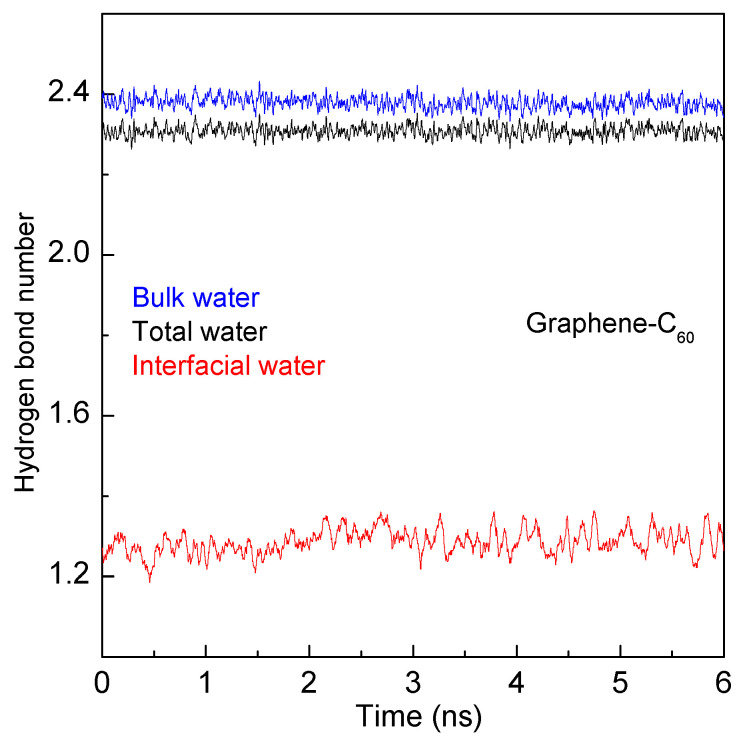
The hydrogen bonding number of interfacial and bulk water when C_60_ fullerene is aggregated with graphene sheet.

In this work, hydrogen bonds for both interfacial and bulk water are calculated based on the geometrical definition [79] using the Visual Molecular Dynamics program [80] (Figure 11). The truncation at the solute–water interface results in interfacial water having fewer hydrogen bonds compared to bulk water. Consequently, the aggregation of solutes in water is driven by the maximization of hydrogen bonding among water molecules.

As an external interface is embedded into water, it may undoubtedly affect the structure of interfacial water. In combination with our recent studies [47,48,49] on hydrophobic interactions, it is found that hydrophobic interactions may undoubtedly be influenced by the external interface. It is found that the interface effects are related to not only the surface molecular polarity but also the geometric characteristics of external interface, such as geometric shape, surface roughness, etc. Regarding the smooth interface, the “attractive” forces are found between solutes and the external interface. These lead to the dissolved solutes tending to be aggregated at the smooth interface.

## 3. Methods

### 3.1. MD Simulations

To investigate the interface effects on hydrophobic interactions, MD simulations were conducted on various systems using the NAMD package [81], such as CH_4_-CH_4_, C_60_-C_60_, graphene–CH_4_, graphene–C_60_, graphene–CH_4_-CH_4_, and graphene–C_60_-C_60_. In the CH_4_-CH_4_ and C_60_-C_60_ systems, one solute was fixed while the other was restrained to move along the *z*-axis toward the fixed solute. In the graphene–CH_4_ and graphene–C_60_ systems, the graphene sheet was fixed, and the CH_4_ molecule or C_60_ fullerene was restrained to move perpendicularly toward the center of the graphene sheet. To investigate the effects of the graphene sheet on hydrophobic interactions in CH_4_-CH_4_ and C_60_-C_60_ systems, the two CH_4_ molecules or C_60_ fullerenes were restrained to move toward the graphene sheet sequentially. In comparison with CH_4_-CH_4_ (C_60_-C_60_) in bulk solutions, these may be used to understand the effects of the graphene interface on hydrophobic interactions when the solutes are associated at the graphene surface.

In this work, the empirical CHARMM force field [82] was used to describe interatomic interactions. The TIP3P water model [83], which is the default in NAMD, was used to represent water molecules. Non-bonded van der Waals interactions were set to zero between 10 and 12 Å. The particle mesh Ewald (PME) algorithm [84,85] was used to account for long-range electrostatic interactions. The simulated temperature was kept at 300 K, employing moderately damped Langevin dynamics. Periodic boundary conditions were applied in the three directions of Cartesian space. Additionally, the equations of motion were integrated with a time step of 2 fs.

### 3.2. PMF Calculations

The MD simulations were performed in the isochoric–isothermal ensemble (NVT). The simulated box was 44 Å × 42 Å × 44 Å. In this work, after the solutes (C_60_, CH_4_) and graphene are fixed, the systems were initially simulated to allow the water molecules to reach thermodynamic equilibrium. Next, the target solutes (C_60_, CH_4_, graphene, graphene–C_60_, graphene–CH_4_) are fixed, the test solute (C_60_, CH_4_) is restrained to move toward the target solutes through Steered MD simulations (Figure 4, Figure 5 and Figure 6). When the solutes are aggregated in water (or the solute is associated with the graphite sheet), PMFs are calculated through US [86,87,88] combined with the Weighted Histogram Analysis Method (WHAM) [89,90,91]. In the US calculations, the separation between solutes (or between solute and graphene) is divided into several windows, about 1 Å width for each window. In this work, using the implementation by Grossfield [92], the WHAM is applied to calculate the PMFs with a tolerance of 10^−7^.

## 4. Conclusions

Building on our recent studies on hydrophobicity, this work investigates the impact of external interfaces on hydrophobic interactions. The following conclusions can be drawn:

(1) The external interface primarily influences the structure of interfacial water and subsequently affects hydrophobic interactions within the system. These effects are linked to both the surface molecular polarity and the geometric characteristics of the external interface;

(2) The impact of the external interface on hydrophobic interactions is reflected in the solutes’ dissolved behaviors in solution. It is observed that solutes tend to aggregate at the external interface rather than within the bulk of the solution;

(3) To maximize hydrogen bonding among water molecules, solutes are likely to accumulate at smooth interfaces. This tendency is influenced by the size and concentration of the solutes.

## Data Availability

Data are contained within the article.
